# Concomitant Syndromic Diagnosis of Mpox and Other Vesicular Viruses in Patients with Skin and Genital Lesions

**DOI:** 10.3390/pathogens13030207

**Published:** 2024-02-27

**Authors:** Maria Beatrice Valli, Antonella Vulcano, Martina Rueca, Giulia Matusali, Valentina Mazzotta, Emanuele Nicastri, Enrico Girardi, Carla Fontana, Andrea Antinori, Fabrizio Maggi

**Affiliations:** 1 Laboratory of Virology, National Institute for Infectious Diseases, Lazzaro Spallanzani IRCCS, 00149 Rome, Italy; mariabeatrice.valli@inmi.it (M.B.V.); martina.rueca@inmi.it (M.R.); fabrizio.maggi@inmi.it (F.M.); 2 Laboratory of Microbiology, National Institute for Infectious Diseases, Lazzaro Spallanzani IRCCS, 00149 Rome, Italy; antonella.vulcano@inmi.it (A.V.); carla.fontana@inmi.it (C.F.); 3 Clinical and Research Infectious Diseases Department, National Institute for Infectious Diseases, Lazzaro Spallanzani IRCCS, 00149 Rome, Italy; valentina.mazzotta@inmi.it (V.M.); emanuele.nicastri@inmi.it (E.N.); andrea.antinori@inmi.it (A.A.); 4 Scientific Direction, National Institute for Infectious Diseases, Lazzaro Spallanzani IRCCS, 00149 Rome, Italy

**Keywords:** mpox, syndromic diagnosis, vesicular lesions, sexual transmission

## Abstract

The recent multi-country outbreak of the zoonotic monkeypox virus (MPXV) infection in humans without an epidemiological link with endemic areas has raised concerns about the route of transmission. Since the infection spread largely among men who have sex with men who, in most cases, presented primary lesions of the genital and oral mucosa, sexual transmission has been proposed. In the present study, we retrospectively evaluated specimens of vesicular lesions collected from the skin and genital tract of 35 patients (23 positive and 12 negative) presenting at our Institute for monkeypox (mpox) diagnosis by using a novel molecular syndromic vesicular virus panel (VVP) assay. All MPXV-positive samples but one was confirmed; however, the viral syndromic analysis revealed that 8.6% of them were coinfected with one or more viruses, and 17% had at least a virus different from the MPXV. The percentage of coinfections increased to more than 25% when nonviral pathogens, such as gonorrhea and syphilis, were also considered. These results show the usefulness of syndromic diagnosis in cases where MPXV is suspected (and vice versa) and at the same time highlight that the broader screening of sexually transmitted infections in the population with high-risk sexual behavior is critical to ensure a complete etiology and appropriate treatment.

## 1. Introduction

Monkeypox is an emerging zoonotic infection caused by the monkeypox virus (MPXV), a species of the genus *Orthopoxvirus*, which includes the variola virus, the causative agent of smallpox [1]. The MPXV has a large genome consisting of double-stranded DNA (dsDNA) of about 200 kb. Based on the geographic isolation of the virus and its genomic characteristics, were identified two distinct clades, namely the Congo Basin (or Central African) clade and the West African clade, which were recently renamed Clade I and Clade II, respectively. The latter includes two subclades, IIa and IIb, to which the virus causing the 2022 outbreak belongs [2,3].

Despite being first isolated in 1958 in monkeys used for polio vaccine research in a laboratory in Copenhagen, Denmark [4], the MPXV has a broad host range and may infect very different species of the lowland tropical forest. However, the natural reservoir is still unknown, although scientific evidence suggests that rodents and nonhuman primates are the most likely [5].

The first human case was described in a child of the Democratic Republic of the Congo in 1970, and since then, MPXV infections have been reported in several parts of Central and West Africa. In these areas, the virus circulates endemically and typically causes small outbreaks, mostly in rural areas where the infections occur from contact with wildlife reservoirs. The virus can also be transmitted from human to human through close contact with infectious material such as skin lesions, body fluids, and respiratory droplets. Several sporadic or small clusters of imported cases of MPXV infection were also reported outside Africa, but all infected individuals had documented histories of recent travel to endemic countries or contacts with infected animals, and secondary spread was limited [6,7]. Therefore, the rapid, widespread, multi-country outbreak of MPXV not epidemiologically linked to endemic areas, which occurred in 2022, raised concerns about the route of interhuman transmission of the virus.

After an incubation period of 5–21 days, the disease typically manifests with clinical biphasic features, with a prodromal phase characterized by fever, myalgia, fatigue, headache, and swollen lymph nodes, often followed by maculopapular rash at the site of the primary infection that can spread to other parts of the body [8]. However, 2022 mpox cases present novel epidemiological and clinical characteristics [9,10,11]. Unlike what was previously described in human mpox infection, the lesions were mainly localized in the genitals rather than on the face, palms, and soles, and they did not show the typical sequential pattern of evolution, from the macula to papula to vesicles to pustules to scab [12]. In addition, the classic progression of the illness with the systemic symptoms preceding cutaneous manifestations was not observed in all patients [12,13].

During the recent outbreak, the spread of the disease was almost exclusively limited to a community of men who have sex with men (MSM), and most of them used to have high-risk sexual behavior such as multiple partners and sex without a condom [9]. Indeed, in a high percentage of cases, MPXV DNA was detected in the genital/anal area of the body, skin, urethral secretion, and seminal fluid, thus indicating that the most likely route of infection was mainly through close contact during sexual intercourse [11]. In particular, the detection of the virus in semen fluid strongly suggests a potential sexual transmission [9].

In addition, the epidemiological data indicate that human-to-human transmission through sexual contact would be the main factor contributing to the recent widespread infection outside the epidemic countries [8,9]. Consistent with this hypothesis, it was found that a high percentage of patients had concurrent sexually transmitted infections [11], and indeed, several cases of coinfection have been reported worldwide [14,15]. Therefore, systematically carrying out a differential diagnosis of sexually transmitted diseases in all cases of suspected MPXV infection is of utmost importance for a proper clinical evaluation and the epidemiology of the disease. In this context, the availability of rapid and accurate diagnostic tools is crucial.

The most widely used diagnostic method is the detection of the viral genome within clinical samples by means of nucleic acid amplification assays (NAATs), which, although more reliable than serological tests due to serological cross-reactivity with other poxviruses, require specialized personnel, dedicated facilities, and various processing steps to obtain results. Several easy-to-use antigen and antibody tests have been developed for a range of specimen types such as serum, cutaneous, and oropharyngeal swabs, which are able to rapidly detect MPXV, but their analytical performance is less sensitive and specific than molecular tests [16]. To overcome the diagnostic limit of antigenic tests, molecular-based point-of-care tests (POCTs), such as those based on CRISPR-Cas12 technology, have been developed [17]. Despite being useful in resource-limited settings, these tests have the limitation of detecting only MPXV but not performing a rapid differential diagnosis or identifying coinfections.

The recent development of syndromic panel-based assays in microbiology has led to a revolutionary improvement in the diagnosis of infectious diseases and clinical management [18]. The main innovative aspect of these multiplex molecular panels is their ability to simultaneously detect several pathogens associated with clinical syndromes, such as meningoencephalitis, gastroenteritis, or respiratory infections, with a rapid response time. Therefore, clinicians can briefly diagnose an infection and make appropriate decisions about the management of the patient, such as isolation, hospital admission, and therapies; thus, resulting in a positive impact on healthcare.

Recently, a new syndromic panel for the viral diagnosis of vesicular rash has been commercialized (QIAstat-Dx Viral Vesicular Panel-VVP; QIAGEN Diagnostics GmbH, Hilden, Germany) [19]. The test is based on a multiplex nucleic acid amplification that allows for the detection of the MPXV and five other viral pathogens that can cause similar skin manifestations. Specifically, the cartridge can identify, in a single reaction, MPXV clade Congo Basin or I (MPXV1), MPXV clade West African or II (MPXV2), herpes simplex virus 1 (HSV1), herpes simplex virus 2 (HSV2), human herpesvirus 6 (HHV6), Enterovirus (EV), and varicella zoster virus (VZV).

Here, we retrospectively tested the specimens of cutaneous lesions collected from the skin and genital districts of 35 patients presenting at the National Institute for Infectious Diseases (INMI) Lazzaro Spallanzani, Rome, for the diagnosis of MPXV DNA with the new molecular syndromic VVP assay to evaluate the performance of this syndromic panel and highlight the presence of eventually viral coinfections.

## 2. Materials and Methods

### 2.1. Procedure

Patients who presented to INMI Lazzaro Spallanzani with acute skin rash, pustules, or mucosal lesions compatible with monkeypox infection underwent a physical examination, and, oropharyngeal, skin, and genital swabs were collected and tested for MPXV DNA by using a commercial *real-time* PCR (ELITechGroup S.p.A., Torino, Italy). Confirmed mpox cases were defined as individuals with positive PCR results. All patients provided written informed consent.

In total, 21 swab samples (11 anal and 10 genital samples) and 15 skin lesions collected from 35 patients who underwent mpox diagnosis from May 2022 to September 2022 were also tested by using the syndromic vesicular virus panel developed by QIAGEN Diagnostics (QIAstat-Dx Viral Vesicular Panel-VVP; QIAGEN Diagnostics GmbH, Hilden, Germany) [19], following the manufacturer’s instructions. This test is a fully automated molecular assay based on a single-use cartridge that includes all the reagents needed for nucleic acid extraction, multiplex real-time PCR (rtPCR) amplification, and the detection of six viruses. The test requires a small sample volume and minimal hands-on time, and the results are available in approximately an hour.

The nucleic acid of all 36 specimens was also extracted by using the QIAsymphony SP instrument (QIAGEN Diagnostics GmbH) and then singularly tested for the presence of the other viruses (EV, HSV1, HSV2, HHV6, and VZV DNA) detected by the VVP assay using virus-specific commercial rtPCRs (AB-ANALITICA, Padua, Italy). These tests were performed simultaneously.

### 2.2. Sequencing and Typing of Enterovirus

The EV-RNA extracted from the positive samples were amplified targeting the partial region of 5′UTR and VP1 gene of enterovirus using Qiagen OneStep RT-PCR (QIAGEN) kit, following the manufacturer’s instructions. The 5′UTR region was amplified using primers described by Nicholson et al. [20], while the amplification of a portion of the VP1 gene was performed using the primers AN88-AN89 developed by Nix et al. [21].

The amplified products were sequenced and analyzed with an ABI prism 3130x1 Genetic Analyzer DNA sequencer; sequences were then aligned and analyzed using BioEdit v 7.0.5.3.

The geno-/serotyping of the Enteroviruses was performed using the web-based open access Enterovirus Genotyping Tool [22] on the basis of the partial sequence of 5′ untranslated region (UTR) for the species assignments and VP1 partial sequences for the serotype assignment.

### 2.3. Microbiological Diagnosis

The detection of sexually transmitted disease-causing pathogens was performed using a commercially available multiplex rtPCR assay (Anyplex II STI-7e; Seegene, Republic of Korea). This assay allows for the simultaneous detection and characterization of target nucleic acids of *Chlamydia trachomatis* (CT), *Neisseria gonorrhoeae* (NG), *Mycoplasma genitalium* (MG), *Mycoplasma hominis* (MH), *Ureaplasma urealyticum* (UU), *Ureaplasma parvum* (UP), and *Trichomonas vaginalis* (TV). The method is suitable for samples collected from genital and extra-genital sites [23]. Nucleic acids were extracted from samples (urine, seminal fluid, pharyngeal, rectal, and vaginal swabs) using the STAR Mag Kit (Seegene) on board the MICROLAB Nimbus system (Hamilton, Reno, NV, USA), which also automates the preparation of the PCR plate using the AnyPlex II STI-7e Kit. Lastly, the rtPCR was performed on a CFX-96 thermocycler (Bio-Rad, Hercules, CA, USA), following the manufacturer’s instructions. The results were interpreted using Seegene viewer software: https://www.seegene.de/software/seegene_viewer, accessed on 3 January 2024.

Patients were subjected to treponemal-specific tests, utilizing a CLIA test for anti-treponemal IgM/IgG (Treponema Screen Kit; DiaSorin, Saluggia, Italy), which was performed on an LIAISON^®^ XL machine (DiaSorin). Positive results were subsequently confirmed using Western blotting, utilizing a Treponema+VDRL ViraBlot^®^ IgG Test Kit and Treponema+VDRL ViraBlot^®^ IgM Test Kit (Viramed Biotech AG, Planegg, Germany), following the instructions issued by the manufacturer.

The detection of *Staphylococcus aureus* was performed using a classical Gram-positive culture system of cutaneous swabs.

## 3. Results

### 3.1. Qualitative Performance of VVP

In this study, 36 vesicular swabs from 35 patients were retrospectively tested using the syndromic VVP assay. The results are shown in Table 1.

Of the 23 samples that tested positive for MPXV DNA in routine diagnostic rtPCR, 22 (96%) were confirmed to be positive for MPXV DNA using the VVP assay. Notably, all the MPXV samples detected belonged to the West African clade. This clade includes both Clade II and Subclade II, recently named Clade III, to which the 2022 epidemic strains belong.

Only one sample yielded a negative result for all viral pathogens. Of the 22 VVP-positive samples, 19 (86%, collected from 18 patients) tested positive for MPXV DNA only, while 3 (14%) showed a coinfection with at least another vesicular virus: Two samples were positive for EV and HSV2 respectively, and one sample was positive for both HSV2 and HHV6. Interestingly, six samples that were identified as negative for MPXV DNA in both rtPCR assays were instead positive for at least one of the vesicular viruses included in the VVP test (i.e., one sample positive for EV RNA, one sample positive for HSV1 DNA, two samples positive for HSV2 DNA, and two samples positive for VZV DNA). Overall, the samples positive for at least one vesicular virus were 28, corresponding to more than 77% of the patients tested.

Importantly, the presence of vesicular viruses other than MPXV revealed by the syndromic VVP assay was confirmed when the samples were tested using the virus-specific rtPCRs.

### 3.2. Quantitative Performance of VVP

**Figure 1:**
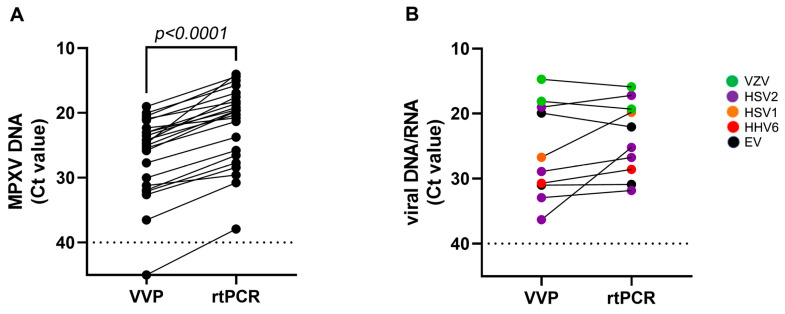
Comparison of cycle threshold (Ct) values obtained with virus-specific real-time PCR (rtPCR) and vesicular virus panel (VVP) test: (**A**) monkeypox virus DNA (MPXV DNA) detection; (**B**) other vesicular viruses’ genome (viral DNA/RNA) detection: varicella zoster virus (VZV), enterovirus (EV), herpes simplex virus 1 (HSV1), herpes simplex virus 2 (HSV2), and human herpes virus 6 (HHV6). Ct comparison was performed using the Wilcoxon test; *p*-values are indicated in each panel. Dot lines represent the limit of the detection of the molecular assays.

To further evaluate the performance of the syndromic VVP test in detecting and quantifying the vesicular viruses, we compared the cycle threshold (Ct) values obtained for each virus using the VVP with those obtained using the virus-specific rtPCR. The comparison was performed by considering the MPXV and the other vesicular viruses separately. Table 1 and Figure 1A show the results of the different Ct values obtained for MPXV detection using the assays, indicating a mean difference of 4.9 Ct (Ct median, IQR: VVP 24.7, 22.3–31.2 Ct; rtPCR 19.7, 17.5–26.5 Ct). This difference can explain the discordant results obtained by analyzing the sample with the highest Ct (37.9), in which MPXV was detected by rtPCR but not by the syndromic VVP test (Table 1).

By contrast, the comparative analysis performed on the other vesicular viruses showed more concordant results, with only two exceptions: One genital swab tested positive for HSV2, and one anal swab tested positive for HSV1 (Table 1 and Figure 1B). However, the number of samples positive for vesicular viruses other than MPXV was too small to allow for an accurate comparative evaluation of the performance of the two tests.

### 3.3. Characteristics of the Patients Tested with the VVP

Table 2 summarizes the epidemiological and clinical characteristics of the individuals tested. The median age was 37 years (IQR 17–61), 94% were male, and 18 (51%) were identified as MSM. Notably, 8 of the 23 positive patients (34.8%) reported known close contact with someone who showed symptoms or had confirmed MPXV infection. Seven patients had traveled within 1 month before symptom onset, but only two of them went abroad. In addition, 12 individuals had HIV (34%), and 14 (40%) reported a previous infection with one or two sexually transmitted pathogens in the last year.

Twenty-seven of the individuals who underwent MPXV DNA diagnosis were also screened for sexually transmitted related pathogens: Four (14.8%) were positive for *Treponema pallidum*, four (14.8%) for *Mycoplasma* (three *M. hominis* and one *M. genitalium)*, three (11.2%) for *Neisseria gonorrhoeae*, two (7.4%) for *Ureplasma urealyticum*, and one (3.7%) for *Chlamydia thrachomatis*. One patient was found to be positive for *Staphylococcus aureus*, a bacterium not included among those that are sexually transmitted. Overall, 18 (56%) of the 35 patients who underwent the mpox diagnosis had an acute infection with one or more viral/bacterial pathogens. Notably, a total of eight mpox cases had a concomitant sexually transmitted infection, indicating coinfection with one vesicular virus in 8.6% (3/35) and with bacteria in 18.5% (5/27) (Table 3).

### 3.4. EV Genotyping

EV species and sero-/genotypes were characterized in the two positive patients through the sequence analysis of the 5′UTR region and the VP1 gene. The genotyping analysis revealed the presence of Coxsackievirus A6 (species A) in the sample from the MPXV-negative patient and of a species D enterovirus in the sample from the MPXV-infected patient. In this latter case, we were not able to determine the EV sero-/genotype due to the low quantity of the virus in the sample (Ct > 35) and the higher variability of the VP1 gene with respect to 5′UTR. However, the genotyping results, revealing two different species, indicate that the two infections were not epidemiologically linked.

## 4. Discussion

In this study, we report the results obtained from our retrospective analysis of the specimens of 35 patients who underwent mpox diagnosis, using a molecular syndromic assay for vesicular viruses. Overall, the syndromic test showed a lower sensitivity for the detection of MPXV DNA compared to individual PCR tests, but it provided a more complex picture of the viral etiology of our cases. Specifically, approximately 50% (6/13) of the negative samples detected by the routine MPXV PCR test were instead positive for at least one vesicular virus other than MPXV; moreover, in three cases (8.6%), the test revealed coinfection with one or two vesicular viruses. Among the vesicular viruses detected, the most frequent were HSV2 (4/36 corresponding to 11% of samples) followed by VZV and EV (2/36; 5.5%), and lastly, HSV1 and HHV6 (1/36; 2.8%). Both HSV1 and HSV2 can cause life-long infections; however, HSV2 is predominantly an anogenital pathogen. Even though the prevalence of genital herpes has decreased globally in the last twenty years, in our cases, HSV2 was the virus most frequently detected [24].

Despite causing a broad spectrum of clinical illnesses, no member of the large family of enteroviruses is properly considered a sexually transmitted virus. However, clinical forms of various skin rashes have been described in acute infections, even if vesicular lesions have been rarely reported except in hand–foot–mouth disease [25]. The enterovirus species in the two cases revealed two different types, suggesting that the infections were not epidemiologically correlated. Notably, one case of EV was caused by Coxsackievirus A6, which is the same serotype recently described as associated with increased circulation in South America and causing an atypical syndrome of hand–foot–mouth disease with cutaneous rush (skin lesions) in unusual parts of the body, including genital areas [26,27]. Enteroviruses are not only transmitted through the oral–fecal route but also through respiratory droplets; therefore, close contact during intercourse likely facilitates disease transmission.

By extending the analysis to a wider spectrum of pathogens, including bacteria known to be sexually transmissible, the proportion of patients with a positive diagnosis of one or more pathogens increased significantly. Although the percentages of nonviral sexually transmitted pathogens were calculated on a slightly reduced number of patients (27 instead of 35), the overall percentage detected was approximately higher than 50%, while that of patients coinfected with MPXV was higher than 25%.

The high prevalence of coinfection with sexually transmitted pathogens in mpox cases found in this study is consistent with previous results [11,14,15] and supports the hypothesis that the MPXV and sexually transmitted infections may share the same route of transmission. This may also explain the recently experienced global spread of infection among a community of sexually active MSM with multiple partners and without protection, even though genomic data on global MPXV sequences pose important questions about the epidemiology of mpox in nonendemic countries as well as the dynamics of the 2022 outbreak.

Phylogenetic studies showed that the genomes of the 2022 strains cluster together, thus suggesting that the outbreak most likely had a single origin. In addition, the cluster (Lineage A1, Clade IIb) is a genetic descendent of the virus that caused the imported cases of mpox from Nigeria in the UK, Israel, and Singapore in 2018 and 2019. Thus, it is likely that the outbreak arose after a prolonged period of cryptic circulation of the imported strain from Nigeria in humans or animals in nonendemic countries. Silent human-to-human transmission may have occurred for years before the outbreak began, which went undiagnosed because several sexually transmitted infections have signs and symptoms that overlap with those of mpox.

The recent outbreak challenges differential diagnosis capabilities and underscores the importance of the systematic screening of sexually transmitted infections in the evaluation of patients with suspected MPXV infection. The main limitation of our study is that we retrospectively tested a small sample population with the syndromic assay; thus, in some cases, clinical and epidemiological data, as well as the diagnostic data of nonviral sexually transmitted infection, were not available. Although complete patient data were lacking, the high percentage of coinfection and infection with viruses other than MPXV highlights the importance of wider screening in the population with high-risk sexual behavior, particularly in cases where MPXV is suspected (and vice versa), to ensure a comprehensive etiology and adequate treatment, such as in the case of coinfection with a herpetic virus.

## Figures and Tables

**Table 1:** Results of 36 samples tested for MPXV DNA using virus-specific real-time PCR and vesicular virus panel test.

Patient	VVP Assay Result	Ct VVP	Ct PCR	Specimen
#1	MPXV2	23.5	19.1	Skin lesion
#2	MPXV2	20.0	15.7	Anal swab
#3	MPXV2	25.0	19.6	Skin lesion
#4	MPXV2	19.0	14.3	Skin lesion
#5	MPXV2	24.3	17.5	Anal swab
#6	MPXV2	24.1	20.2	Skin lesion
#7	MPXV2	30.0	25.7	Anal swab
#8	MPXV2	31.2	29.5	Skin lesion
#9	MPXV2	31.9	26.5	Skin lesion
#10	MPXV2	24.7	14.0	Anal swab
#11	MPXV2	20.5	14.9	Anal swab
#12	MPXV2	22.3	20.7	Anal swab
#13	MPXV2	36.5	30.7	Skin lesion
	MPXV2	32.6	28.3	Genital swab
#14	MPXV2	32.1	27.7	Genital swab
#15	MPXV2	21.1	16.8	Genital swab
#16	MPXV2	23.0	19.5	Genital swab
#17	MPXV2	20.9	18.2	Anal swab
#18	MPXV2	27.7	23.7	Genital swab
#19	Negative	NA	37.9	Genital swab
#20	MPXV2	23.0	18.5	
	EV	31.0	30.8	Skin lesion
#21	MPXV2	25.8	19.7	
	HSV2	36.3	25.1	Genital swab
#22	MPXV2	25.4	21.2	
	HSV2	32.9	31.8	Anal swab
	HHV6	30.7	28.5	
#23	EV	19.9	22.0	Skin lesion
#24	HSV1	26.7	19.8	Anal swab
#25	HSV2	28.9	26.7	Genital swab
#26	HSV2	19.0	17.2	Anal swab
#27	VZV	14.7	15.8	Genital swab
#28	VZV	18.1	19.2	Skin lesion
#29	Negative	NA	NA	Skin lesion
#30	Negative	NA	NA	Skin lesion
#31	Negative	NA	NA	Genital swab
#32	Negative	NA	NA	Skin lesion
#33	Negative	NA	NA	Skin lesion
#34	Negative	NA	NA	Anal swab
#35	Negative	NA	NA	Genital swab

List of abbreviations: NA, not applicable for negative result; MPXV2, monkeypox virus Clade II; HSV1, herpes simplex virus 1; HSV2, herpes simplex virus 2; EV, Enterovirus; HHV6, human herpes virus 6; VZV, varicella zoster virus.

**Table 2:** Epidemiological and clinical characteristics of the study population.

Characteristics	Patient (35)
Age, median (IQR)	37 (17–61)
Gender (%)	
Male	33 (94%)
Female	2 (6%)
Sexual Orientation	
MSM	18 (51%)
Heterosexual	1 (3%)
Unknown	16 (46%)
Transmission Route	
Sexual close contact	17
Household	1
Unknown	17
HIV	
Positive	12
Negative	16
Unknown	7
Recent Travel	
Yes	7
No	12
Unknown	19
Previous STI (Last Year)	
*Treponema pallidum*	12
*Neisseria gonorrhoeae*	1
*T. pallidum* and *N. gonorrhoeae*	1
Negative	3
Unknown	18
Confirmed STI or Vesicular Virus	18 (54%)
*Treponema pallidum*	4
*N. gonorrhoeae*	3
*Mycoplasma* spp.	4
*Ureaplasma urealyticum*	2
*Chlamydia trachomatis*	1
Other bacteria	1
EV	2
HSV 1	1
HSV2	4
HHV6	1
VZV	2
Negative or unknown	17 (46%)
Concomitant STI or viral infection–Nr/MPXV positive	8/22 (36%)

List of abbreviations: MPXV, monkeypox virus; HSV1, herpes simplex virus 1; HSV2, herpes simplex virus 2; EV, Enterovirus; HHV6, human herpes virus 6; VZV, varicella zoster virus; STI, sexually transmitted infection.

**Table 3:** Results of viral and microbiological sexually transmitted infections in patients tested by VVP assay. The vesicular viruses include those detected by the syndromic VVP test: herpes simplex virus 1, herpes simplex virus 2, enterovirus, human herpes virus 6, and varicella zoster virus.

Patients Positive:	All Patients (n = 35)	%
MPXV	13	37
MPX and vesicular virus	3	8.6
Vesicular virus	6	17
MPXV and bacterial STI	5/27	18.5 *
Bacteria STI	4/27	14.8 *
Other bacteria	1/27	3.7 *
**Patients negative**	3 (2 not tested for STI)	

List of abbreviations. MPXV, monkeypox virus; STI, sexually transmitted infection; * percentage calculated considering only the twenty-seven patients known to have undergone microbiological screening.

## Data Availability

The data presented in this study are available upon request from the corresponding author.
